# General Therapeutics and Materia Medica, Adapted for a Medical Text-Book

**Published:** 1854-01

**Authors:** 


ART. VII.—*General Therapeutics and Materia Medica, adapted for a Medical Text-Book.* By ROBLEY DUNGLISON, M. D., Professor, etc., etc. Fifth edition, revised and improved. Two vols. Philadelphia: Blanchard and Lea. 1853.

We have expended some time in looking over and perusing those portions of this work, which would be likely to contain expressions of opinion which might furnish the usual matter for a review of its merits. As a text-book for students, it possesses many valuable qualities. Its arrangement is not too complex, its descriptions are clear and concise, and it is not very much overloaded with theory.

As a reviewer, we look upon a book with different eyes than as a practitioner. The reviewer likes to see some salient points, some distinctive doctrines to serve as landmarks, and which may give tone and character to the book. Such a point we thought we had found in Dr. Dunglison's remarks upon the *vis medicatrix naturæ.* On page 37, speaking of recuperative power, he says: "The existence, then, of such an instinctive power can neither be denied nor lost sight of in the treatment of disease. The error has been, that undue weight has been attached to it, so that the practitioner was guided by its manifestations—or fancied manifestations—in laying down his indications of cure; and if no such manifestations existed, he waited vainly—and too often unfortunately—until the time had, perhaps, gone by for the successful administration of efficacious agents. * * * * There are but few cases, however, in which trust can be safely placed in such a power."

In the foregoing extract would be some matter for the critic, were he not saved the labor by the perusal of succeeding chapters. It is, perhaps, a hypercriticism to say, that Dr. Dunglison is too little liable to the attacks of the critic. The sum of this idea may be recognized, in the fact that the advocate of theoretical medicine, the opponent of statistics, and the veriest numeralist in the French School of Observation, may alike quote this work as supporting their peculiar views.

This is easily enough understood by those accustomed to medical writing, and that without imputing to Dr. Dunglison any time-serving proclivities. In writing of medical theory, he reasons from agriculture and the arts as to the necessity of theory. When he comes to the investigation of imaginative cures, of homœopathy, and of hygienic regimen, he gives to the facts as they present themselves their due importance, and ascribes to nature a very large share in the curative process. We submit that "what is sauce for the goose, is sauce for the gander."

We should like to see a work on Materia Medica from M. Louis, or Elisha Bartlett. The teacher of materia medica naturally magnifies the powers of the agents he handles. Louis, or Bartlett, being strict empiricists, (in the true sense of that misused word,) would be apt to require more exact and definite proof of the action of drugs. They would be perpetually questioning between "*post hoc*," and "*propter hoc*." Undoubtedly they might carry the matter too far, but what they did assert as proved, we might believe confidingly.

"*Marcus dixit, ita est*," might be applied to them without satirical meaning.

